# Monitoring the age-specificity of measles transmissions during 2009-2016 in Southern China

**DOI:** 10.1371/journal.pone.0205339

**Published:** 2018-10-08

**Authors:** Ka Chun Chong, Pei Hu, Steven Lau, Katherine Min Jia, Wenjia Liang, Maggie Haitian Wang, Benny Chung Ying Zee, Riyang Sun, Huizhen Zheng

**Affiliations:** 1 JC School of Public Health and Primary Care, The Chinese University of Hong Kong, Hong Kong, China; 2 Clinical Trials and Biostatistics Laboratory, Shenzhen Research Institute, The Chinese University of Hong Kong, Hong Kong, China; 3 Center for Disease Control and Prevention of Guangdong Province, Guangzhou, China; Australian National University, AUSTRALIA

## Abstract

**Background:**

Despite several immunization efforts, China saw a resurgence of measles in 2012. Monitoring of transmissions of individuals from different age groups could offer information that would be valuable for planning adequate disease control strategies. We compared the age-specific effective reproductive numbers (*R*) of measles during 2009–2016 in Guangdong, China.

**Methods:**

We estimated the age-specific *R* values for 7 age groups: 0–8 months, 9–18 months, 19 months to 6 years, 7–15 years, 16–25 years, 26–45 years, and ≥46 years adapting the contact matrix of China. The daily numbers of laboratory and clinically confirmed cases reported to the Center for Disease Control and Prevention of Guangdong were used.

**Results:**

The peak *R* values of the entire population were above unity from 2012 to 2016, indicating the persistence of measles in the population. In general, children aged 0–6 years and adults aged 26–45 years had larger values of *R* when comparing with other age groups after 2012. While the peaks of *R* values for children aged 0–6 years dropped steadily after 2013, the peaks of *R* values for adults aged 26–45 years kept at a high range every year.

**Conclusions:**

Although the provincial supplementary immunization activities (SIAs) conducted in 2009 and 2010 were able to reduce the transmissions from 2009 to 2011, larger values of *R* for children aged 0–6 years were observed after 2012, indicating that the benefits of the SIAs were short-lived. In addition, the transmissions from adults aged between 26 and 45 years increased over time. Disease control strategies should target children and adult groups that carry high potential for measles transmission.

## Background

Measles is a highly contagious acute viral disease. Throughout the world, and most countries have set goals for its elimination. In 1978, the national Expanded Program on Immunization (EPI) in China started to implement a standard schedule for the routine administration of one dose of measles-containing vaccine (MCV1) among children between 8 and 24 months of age. Subsequently, the mean annual measles incidence decreased from 355 per 100,000 in 1970–1979 to 53 per 100,000 in 1980–1989 [1]. In 1986, a two-dose routine measles immunization program was implemented for children aged between 8 months and 7 years. The age schedule for the second dose of MCV (MCV2) was shifted to 18–24 months in 2005. During 2000–2009, the number of measles cases showed a remarkable decrease but remained around 6.8 per 100,000 on average [2]. In 2006, the government of China set a goal to eliminate measles by 2012, for which purpose a series of programs was implemented, including strengthening routine immunization surveillance, supplementary immunization activities (SIAs), and case-based surveillance [2]. An SIA is defined as the administration of a supplementary dose of a vaccine to a specific age population in a certain area during a short period, regardless of the recipients' previous vaccination histories. SIAs enhance routine immunization programs, including catch-up campaigns, follow-up campaigns, and outbreak-response immunization. The estimated coverage rate of routine immunization with MCV1 increased from 80.4% in 2000 to 91.1% in 2009, whereas the estimated coverage rate for MCV2 was <80% before 2005 and 84.3% in 2009 [1]. In September 2010, China conducted a synchronized, nationwide SIA that targeted children aged 8 months to 14 years, covering 102 million children with a reported coverage rate of 97.5% [1–3]. Although the annual measles incidence had dropped to 0.46 per 100,000 in 2012, it resurged to more than 1.96 per 100,000 in 2013 [3]. Despite the implementation of two-dose routine vaccines since 2005, frequent outbreaks have occurred over the past years [4–6].

**Fig 1:**
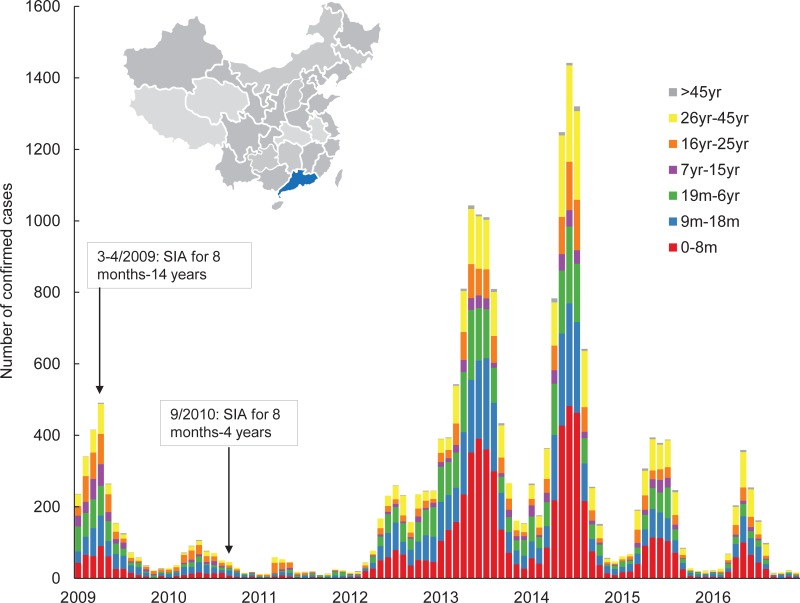
Location of Guangdong Province in China and monthly number of reported cases of measles and immunization activities in Guangdong Province from 2009 to 2016. In 2009, a province-wide SIA was administered to children aged 8 months to 14 years old in Guangdong during March to April. In 2010, another SIA for children aged between 8 months and 4 years old was administered in the province during September.

Guangdong, a highly populated province with 108 million population in 2015, is located in the southernmost part of mainland China (Fig 1). The incidence of measles in Guangdong dropped to a remarkable low in 2011 (0.30/100,000) after a series of SIAs targeting children aged 8 months to 14 years during 2009 and 2010. However, Guangdong had the highest number of reported cases in China in 2012 and 2013, with incidences of 1.84 and 6.64 per 100,000, respectively [7], even though the reported coverage of MCVs was kept above 98% every year after 2009. To this end, the Guangdong government implemented some mop-up vaccination programs after 2012 targeting children aged 8 months to 6 years (Fig 1).

Monitoring the effectiveness of the measles control policy is done by surveillance. In China, measles is a category B infectious disease which indicates it is highly contagious and must be reported to the surveillance system within 24 hours after confirming the laboratory samples [8]. Since 2004, China has a direct network reporting system and automatic warning information system for infectious diseases. The system focusses on the number of reported cases, but does not evaluate the transmissibility of measles.

The effective reproduction number, *R*, is a key epidemiologic variable that summarizes the transmissibility of infectious diseases. It is defined as the expected average number of secondary cases produced by an infectious individual in a population in which not all the individuals are susceptible [9]. When *R* is larger than 1, an infectious individual is expected to infect more than one secondary case. When *R* is less than 1, an infectious individual tends to infect less than one secondary case, and the incidence will decrease. Nevertheless, some infectious diseases have been shown to be strongly age-specific, for example, measles. Age-specific *R*, defined as an average total number of secondary cases from all age groups generated by a single case with respect to his age group was recommended to study the differences in transmission potential taking account of social mixing [10–15]. Although a usual interpretation of age-specific *R* for gauging the control measures required to eliminate an infection is inappropriate [16, 17], age-specific *Rs* provide valuable information on the underlying heterogeneous transmission between and within different groups of individuals. For example, Glass et al. estimated the *R*s of pandemic influenza A(H1N1) for children and adults and identified children had a higher transmission then adult cases.

In China, the demography of measles infections has changed over time. While infants aged between 9 months and 18 months and young adults aged 16 to 25 years were the primary population of measles infections, children aged 0 to 8 months and adults aged 26–45 years became the primary sources after the national SIA. Apparently, the age specificity in measles transmissions could be affected by vaccination policies. Chong et al. [18] showed that even though substantial decreases in the numbers of cases were observed after mass vaccination campaign, measles could still persist in a population given a high value of *R*.

The majority of the relevant epidemiological studies conducted in China have been based on reported cases and have aimed to describe the incidence and characteristics of population distribution [19, 20]. However, the age specificity in measles transmissions had hardly been studied. In the present study, we compared the age-specific *R* of measles infections between different age groups by using laboratory and clinically confirmed data collected from 2009 to 2016.

## Methods

### Data collection

Daily notifications of measles cases from January 1, 2009 to December 31, 2016 were collected from the National Infectious Disease Monitoring Information System (NIDMIS), as complied by the Center for Disease Control and Prevention (CDC) of Guangdong Province. For some of the cases with typical clinical symptoms, case notifications were sent to the person in charge of reporting by outpatient or resident doctors, and the cases were then recorded as “clinically confirmed”. Blood samples from these cases were sent to CDC clinical laboratories for confirmation, if laboratory capacity allowed. Other cases with atypical clinical symptoms were recorded as “suspected cases,” and the blood samples of these cases were subsequently transferred to a diagnostic laboratory to obtain a confirmed diagnosis. The test results were returned to the patients’ doctors. If the test results were positive, the cases were relabeled as “laboratory-confirmed cases,” and the person in charge of reporting was notified. If the results were negative, the cases were relabeled according to the specific disease that had been detected before handing over to the reporting personnel. For the (clinically confirmed or suspected) cases without laboratory confirmation, epidemiological investigations were conducted to determine whether the patients’ infections had any linkage to other confirmed cases within 7–21 days before the onset of any symptom. The epidemiological investigations were performed through direct contacts in the relevant village, community, or school, or through direct contacts for mass gathering events. The clinically confirmed and laboratory-confirmed cases were both regarded as cases, and reporting personnel were required to report such cases to the NIDMIS within 6 hours.

We divided the population into 7 age groups according to the age of onset: 0–8 months (pre-vaccination age), 9–18 months (received the first dose of the measles containing vaccine, MCV-1), 19 months to 6 years (received the second dose of the measles containing vaccine, MCV-2), 7–15 years (primary and secondary school students), 16–25 years (high school and college students/young adults), 26–45 years (mature adults), and ≥46 years (aged adults).

This study was reviewed and approved by the Medical Ethics Committee of the Guangdong CDC. The application of the data in this study has been authorized by the Guangdong CDC. All data were fully anonymized prior to access by any of the authors and does not involve patients' privacy prior collection. Informed consents were exempt from the ethics committee in accordance to the CDC policy of continuing public health investigations of notifiable infectious diseases, in which the patient names, addresses, medical histories with infectious diseases, and their family information will not be disclosed to the public by Guangdong CDC in any form. The data are available without restrictions (the link will be provided after the acceptance of the paper).

### Statistical methods

The method for estimating the age-specific effective reproduction numbers suggested by White et al. [10], which is a modification of the Wallinga and Teunis approach [21], was adopted. Let *p*_*d*_ denote the probability of a serial interval of length *d*, (*d* = 1,2,…,*D* where *D* be the maximum serial interval length), $n_{t,g_{i}}$ denote the frequency of symptom onset in age group *g*_*i*_ (*i* = 1,2,…,*G* where *G* is the total number of age groups) on day *t* (*t* = 1,2,…,*T* where *T* is the length of the study period), $r_{g_{i}\to g_{j}}$ denote the contact rate between two individuals from age group *g*_*i*_ and age group *g*_*j*_ (*j* = 1,2,…,*G*). The effective reproduction number of age group *g*_*i*_ on day *t*, $R_{t,g_{i}}$, can be calculated by summing the expected number of individuals in each age group from *t+1* to *t+D* infected by an individual in age group *g*_*i*_ whose symptom onset was on day *t*:

$$R_{t,g_{i}}=\{\begin{array}{cc} \sum_{d=1}^{\min(D,T-t)}\sum_{j=1}^{G}n_{t+d,g_{j}}\times P(I_{t,g_{i}}\to I_{t+d,g_{j}}) & t\neq T\\ 0 & t=T \end{array}$$

where

$$P(I_{t,g_{i}}\to I_{t+d,g_{j}})=\{\begin{array}{cc} \frac{r_{g_{i}\to g_{j}}\times p_{d}}{\sum_{k=1}^{\min(t+d-1,D)}\sum_{l=1}^{G}n_{t+d-k,g_{l}}\times r_{g_{l}\to g_{j}}\times p_{k}} & n_{t,g_{i}}\neq 0\\ 0 & n_{t,g_{i}}=0 \end{array}$$

denote the relative probability that an individual in group *g*_*j*_ on day *t*+*d* was infected by an individual in group *g*_*i*_ on day *t*.

In this study, there were 7 age groups (i.e., *G* = 7) and the maximum serial interval length *D* was set at 20. *p*_*d*_ was generated from a gamma distribution with a mean of 7 days and standard deviation of 3 days [14]. $r_{g_{i}\to g_{j}}$ was estimated by using the contact matrix of China, projected by the Bayesian hierarchical model in Prem et al [22]. The estimation formulas were implemented in Microsoft Excel.

### Uncertainty generation

We extended the probabilistic method described in White et al. to generate the statistical uncertainty [15]. A parametric bootstrapping approach was employed to generate 1,000 realizations of $\left\{R_{t,g_{i}}\right\}$. In each iteration, we generated a new dataset by first simulating the total number of individuals in group *g*_*j*_ (*j* = 1, 2, …, 7) infected by those in group *g*_*i*_ (*i* = 1, 2, …, 7) with symptom onset of day *t* (*t* = 1, 2, …, *T*-1) from a Poisson distribution with mean = $n_{t,g_{i}}\times \sum_{d=1}^{\min(D,T-t)}n_{t+d,g_{j}}\times P(I_{t,g_{i}}\to I_{t+d,g_{j}})$, which can be interpreted as the estimated total number of individuals in group *g*_*j*_ infected by all the individuals in group *g*_*i*_ with symptom onset on day *t*, where $n_{t,g_{i}}$ and $P(I_{t,g_{i}}\to I_{t+d,g_{j}})$ were directly obtained and calculated from the original dataset respectively. The simulated number was then distributed within the serial interval *t* + 1 and *t* + 20 according to a Gamma distribution with a mean of 7 days and standard deviation of 3 days. The above procedure was repeated for all *i*,*j*, and *t*. The resulting data were used to calculate a realization of $\left\{R_{t,g_{i}}\right\}$ and further averaged by months. The 95% credible intervals (CI) of the monthly estimates were obtained from the 2.5^th^ and 97.5^th^ percentiles over the 1,000 realizations.

### Sensitivity analysis

Apart from China’s contact matrix, contact matrixes from 8 other countries were employed to test the sensitivity of our results [23]. We also tried evaluating the estimates using 12 days and 3 days as the mean and standard deviation of the gamma distribution of the serial interval [24].

## Results

**Fig 2:**
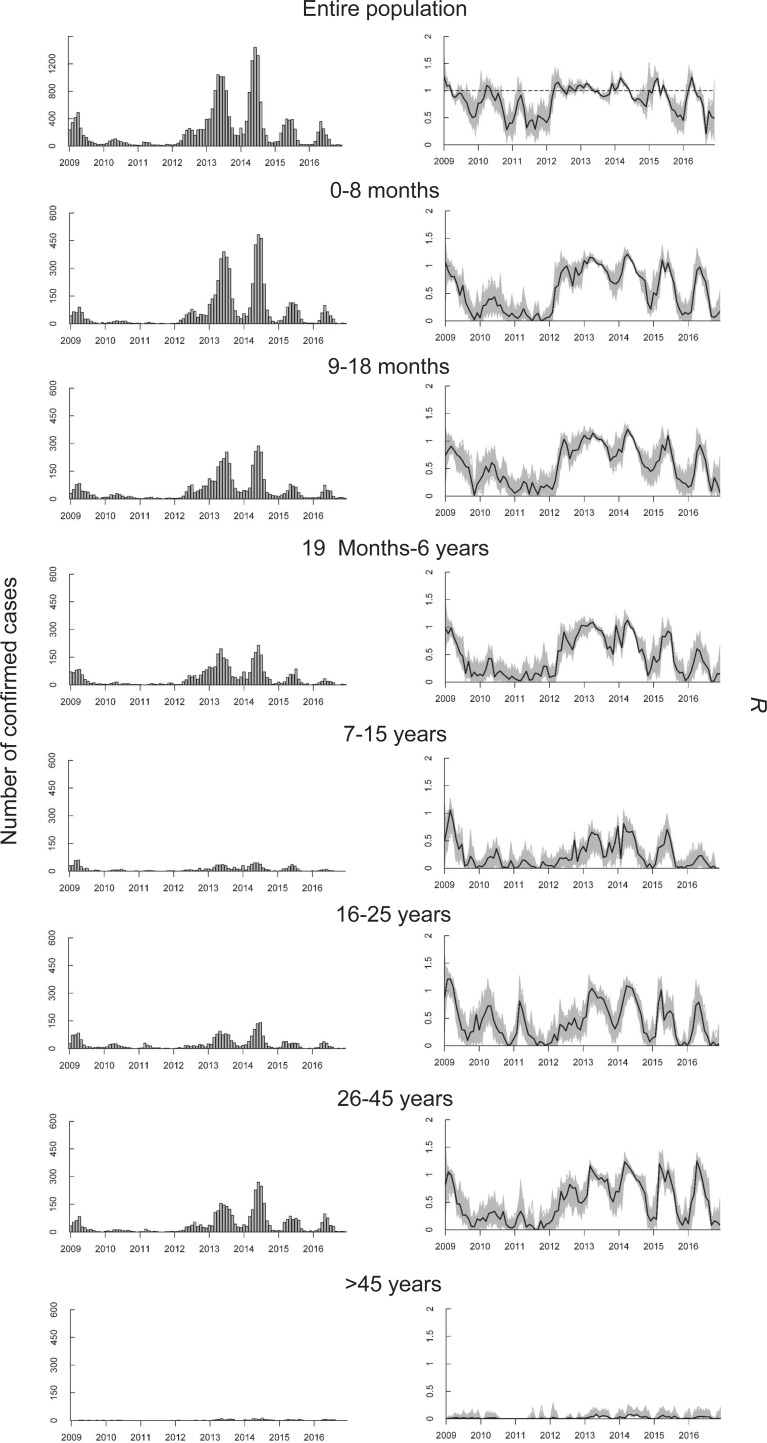
Monthly number of reported cases (left panel) and the estimated effective reproductive numbers (black line in right panel) with 1,000 realizations (grey lines in right panel) for the entire population and 7 age groups.

Fig 2 presents the estimated age-specific effective reproductive numbers. In general, the peak *R* values of the entire population were 1.16 (95% CI: 1.11 to 1.30), 1.09 (95% CI: 1.04 to 1.11), 1.24 (95% CI: 1.19 to 1.31), 1.23 (95% CI: 1.15 to 1.29), and 1.25 (95% CI: 1.11 to 1.31) from 2012 to 2016 respectively, indicating the persistence of measles in the population. Across all age groups, the *R* values increased greatly from 2009–2011 to 2012–2016, particularly for those of children under 7 years old (the first 3 age groups) and adults aged between 26 and 45 years.

The estimates for children aged 0–6 years (the first 3 age groups) shared a similar tendency throughout the study period. After 2012, the *R* values started to increase, earlier than in the other groups, which indicated that children aged 0–6 years had a large contribution in disease transmissions of the measles outbreaks (the peaks of *R*_*t*,0−8*m*_ = 1.15 (95% CI: 1.10 to 1.22), *R*_*t*,9−18*m*_ = 1.15 (95% CI: 1.09 to 1.18), *R*_*t*,19*m*−6*y*_ = 1.09 (95% CI: 1.03 to 1.12) in 2013). The *R* peaked at remarkably different points in regard to the maximum numbers of cases. Nevertheless, the *R* values for these 3 groups gradually decreased from 2014 to 2016 (i.e. estimated peak values respectively dropped from *R*_*t*,0−8*m*_ = 1.16 (95% CI: 1.03 to 1.28), *R*_*t*,9−18*m*_ = 1.21 (95% CI: 1.14 to 1.27), *R*_*t*,19*m*−6*y*_ = 1.13 (95% CI: 1.07 to 1.18) in 2014, to *R*_*t*,0−8*m*_ = 0.98 (95% CI: 0.92 to 1.11), *R*_*t*,9−18*m*_ = 0.93 (95% CI: 0.84 to 1.08), *R*_*t*,19*m*−6*y*_ = 0.60 (95% CI: 0.32 to 0.76) in 2016), indicating the measles transmissions from children aged under 7 years declined over time.

The *R* values estimated for children aged 7–15 years were low across the study period in general, even though the values also increased since 2012, indicating that primary and secondary school students had a limited contribution to measles transmissions. Similarly, the results for the adults aged ≥46 years were extremely low across the study period, indicating that these persons were unlikely to infect more than a case on average.

The *R* values of young adults aged 16 to 25 years had several distinct peaks from 2009 to 2011, which were different from those of other age groups. After 2012, the *R* values steadily increased with annual peaks of 1.04 (95% CI: 0.84 to 1.12), 1.09 (95% CI: 0.86 to 1.17), and 1.01 (95% CI: 0.71 to 1.18) in 2013, 2014, and 2015, respectively.

For adults aged between 26 and 45 years, a clear seasonal pattern of *R* values was observed after 2012, which showed a similar trend to that observed in children. In 2014 and 2015, the estimated annual peaks of *R* were 1.24 (95% CI: 1.03 to 1.33) and 1.20 (95% CI: 1.01 to 1.39) respectively. Given that the *R* peak of the entire population was significantly above unity in 2016, adults aged 26–45 years had the largest contribution to measles transmissions.

S1 Fig shows the sensitivity of the results to the use of contact matrices from other countries [23]. In general, the major findings were robust with the variation of contact patterns, for example, children aged 0–6 years still had a large contribution in measles transmissions after 2012. Nevertheless, due to a difference in contact frequency, larger estimates were observed for children aged 0–8 months and 9–19 months when using the contact matrix of Germany. Moreover, while using Poland's contact matrix drew a lower estimates for children aged 0–8 months and 9–19 months, it drew slightly larger estimates for adults aged 16–25 years and 26–45 years after 2012.

We also investigated using a different set of parameters for the distribution of the serial interval, and found that the results were generally consistent with the main analysis (S2 Fig). The *R* values of children groups were slightly increased, whereas the *R* values of adult groups were slightly decreased.

## Discussion

Monitoring the age specificity of measles transmissions could provide information that would be valuable to officials who seek to develop adequate disease control strategies. For example, it could help to select appropriate age groups for supplementary vaccination. In this study, we compared the age-specific *R* of measles infections between different age groups, using laboratory and clinically confirmed data from 2009 to 2016 for Guangdong Province. According to the results, measles transmissions varied across most age groups before and after 2012 and the large values of *R* from the entire population indicated a persistence of measles in the population from 2012 to 2016. In general, children aged 0–6 years and adults aged 26–45 years had higher contributions in measles transmissions when comparing with other age groups after 2012. After 2013, while the peaks of *R* values for children aged 0–6 years dropped steadily by years, the peaks of *R* values for adults aged 26–45 years remained unchanged and kept at a high range every year, demonstrating the highest contributions in measles transmissions. The findings suggest that disease control strategies should target children and adult groups that carry a high potential for measles transmission.

As has been previously noted, we found that children aged 0–6 years had R values that increased after 2012, even though SIAs targeted this population in 2009 and 2010. The increasing R values could have resulted from low MCV coverage in this cohort, for which the official reported coverage was usually over-estimated [25, 26]. An in-house survey of a similar cohort of children aged 24–47 months showed that MCV1 and MCV2 coverage rates were only 83% and 75%, respectively [25], results that were inconsistent with the generally reported figure of >98% in China [27]. The geographic heterogeneity of vaccine coverage in China could be another explanation [28, 29]. A Chinese study indicated that the measles antibody levels of children aged 2–10 years old were significantly lower for residents of rural areas than for residents of urban areas [28]. The primary reasons why rural children had missed their MCVs were because they were living far from the clinics and because they were unable to access vaccination information [30]. The incomplete immunization records of rural children also made it more difficult for public health officials to track them in order to administer the vaccine.

We observed elevated transmissions in infants aged 0–8 months, which may primarily be attributed to the design of the immunization system, which regarded them as too young to be vaccinated by either routine immunization or SIAs. A longitudinal study of maternal measles antibody titers in infants in Guangzhou (the provincial capital of Guangdong Province) showed that titers among infants decreased rapidly after 3 months of age, and were generally undetectable at 7 months of age [31]. Several other studies reported similar results [32, 33]. Hence, there was a remarkable immunity gap among children under 8 months old. Some studies showed that only around 2.7% to 6.8% of infants are seropositive for measles at 6 months of age [34, 35]. Nevertheless, even though infants aged 0–8 months were identified as a high transmissibility group, reducing the minimum age for receiving MCV-1 to 6 months is controversial. We also found that the transmissions from children aged 7–15 years were comparatively low, which was expected given that given that they were the main target of previous SIAs. Moreover, many primary schools implemented screening of children’s vaccination certificates and administered supplementary doses of the measles vaccine to fill immunity gaps before the annual entrance [36].

The values of age-specific *R* for adults aged between 26 and 45 years kept at a high range from 2013 to 2016 and it could be attributed to the lower efficacy of measles vaccines, the low vaccination coverages during 1980s and earlier, and the reduced chance of natural infections. Persons aged 26–45 years at the time of the present study were thought to be the first recipients of the vaccination after the approval of routine immunization. Liquid vaccines were used for immunization at that time. They had a lower effective dosage and may have resulted in the lower level or shorter protective duration of antibodies among the population. In addition, a functional cold chain, transportation, and communication system for the measles vaccine had not been established at that time; hence, the quality and efficacy of the vaccines could not be guaranteed. Secondly, several parents knew nothing about the measles vaccine and underestimated the severity of measles, thereby resulting in a low vaccination rate and a high rate of unsure inoculation history. Thirdly, secondary vaccine failure (i.e. measles onset after vaccination and successful seroconversion) due to waning immunity might have occurred among vaccinated adults. Although our study could not identify secondary vaccine failure from other cases as serological evidence of previous successful vaccination were lacked, it has been concluded by WHO that waning immunity has not played a major role in the transmission of measles compared to the absence of initial immunity [37]. The proportion of cases attributable to secondary vaccine failure varied greatly across outbreaks [38]. In a cohort study (n = 2882) in Zhuji County of Zhejiang province, around 11–13% of those given with single doses of domestic vaccines would become sero-negative (measured by haemagglutination-inhibition tests) after 14 years, yet clinical measles cases rarely happened among them who had humoral immunity waned as they were still protected by secondary immune response [37,39]. Finally, the subsequent SIAs did not cover these persons; thus, the immunity gaps among people aged 26 to 45 years increased.

On the other hand, the transmissions from individuals aged ≥46 years were the lowest among the age groups studied, even though there was almost no vaccination history in this group. We believe that the majority of these individuals acquired antibodies through natural infection, owing to the highly contagious nature of measles when they were young. Moreover, many studies have shown that seropositivity after natural infection persists longer and generates a stronger response than the immunity acquired from vaccination [39–41].

Given the increasing values of *R* for the entire population observed after 2012, some mop-up vaccination campaigns in 2012 and 2013 appear to have had limited effectiveness, even though they aimed to control measles transmission. One reason for this is that rural families usually have a lower level of education and do not fully understand information regarding mop-up campaigns, which results in a lack of initiative to get the vaccine. Moreover, some of the susceptibles were migrants, and officials reported difficulties tracking their vaccination histories. Although door-to-door notifications, text messages, and telephone notifications have been used to inform migrant families to join mop-up campaigns [42], it is often difficult to contact these families because of changes to their addresses or phone numbers.

From a policy-making perspective, these results imply that for a successful measles control campaign, the public health department should carry out control measures for appropriate age groups. For children between 9 and 18 months old, it is necessary to take measures to improve vaccination coverage, including providing more publicity to improve parents’ awareness about vaccination against measles, creating integrated multichannel notifications to inform parents of the vaccination, and strengthening the supervision of kindergartens. For adjustments to the immunization strategy, adult-specific vaccination programs should be considered to fill the immunity gaps among adults, especially for those aged between 26 and 45 years.

One of the major limitations in this study is the quality of notification data. Indeed, a proportion (~30%) of the notification data in the early phase study (2009–2011) was only clinically confirmed which may lead to some misdiagnosis as well as an underestimate of the age-specific *R*s. Nevertheless, more than 95% of cases were laboratory confirmed after 2011. Particularly, the reliance on clinically confirmation was more in rural hospitals in which doctors may lack sufficient knowledge on measles diagnosis. The positive predictive value of a clinical definition would also be changed over time as measles has become rarer by time. To minimize the chance of misdiagnosis, the clinically confirmed cases were not only identified by clinical symptoms, but were also investigated with any potential epidemiological association with other laboratory-confirmed cases. Moreover, the completeness of the data could be affected by underreporting as some of the parents might have regarded measles as a kind of skin disease or might have confused it with other diseases that involve skin rashes. Apart from that, age-specific *R*s are common to be used as a metric to identify appropriate age groups most responsible for transmission as for a target of interventions [10, 12, 13]. However, when determining the effort required to eliminate an infection, the interpretation of an age-specific *R* is different from that of an overall *R* in a heterogeneous population as using the original threshold of unity could lead to an underestimation of target population for interventions [16, 17]. Alternatively, Roberts & Heesterbeek [17] suggested a type-reproduction number which can single out particular subgroup rather than averaging over all subgroups. Further works such as generalizability and statistical inference [16] on the alternative measures worth being investigated.

## Conclusions

In summary, we compared the age specificity in measles transmissions from 2009 to 2016 in Guangdong Province. Although the provincial SIAs conducted in 2009 and 2010 were able to reduce the transmission rates from 2009 to 2011, larger effective reproductive numbers for children aged 0–6 years were observed after 2012, which indicates that the benefits of the SIAs were short-lived. In addition, the transmissions from adults aged between 26 and 45 years increased over time. Based on the findings of the present study, we believe that disease control measures should strategically target those groups that carry a high potential for measles transmissions.

## Supporting information

S1 Fig Effective reproductive numbers for 7 age groups in 8 countries with different contact matrices. (PDF)

S2 Fig Monthly number of reported cases (left panel) and the estimated effective reproductive numbers (black line in right panel) with 1,000 realizations (grey lines in right panel) for the entire population and 7 age groups, using 12 days and 3 days as the mean and standard deviation of the gamma distribution of the serial interval.
(PDF)
