# Supplementary figures and images for: Monitoring the age-specificity of measles transmissions during 2009-2016 in Southern China
Source: PLoS One. 2018 Oct 8;13(10):e0205339. doi: 10.1371/journal.pone.0205339 (PMC6175510; doi:10.1371/journal.pone.0205339)

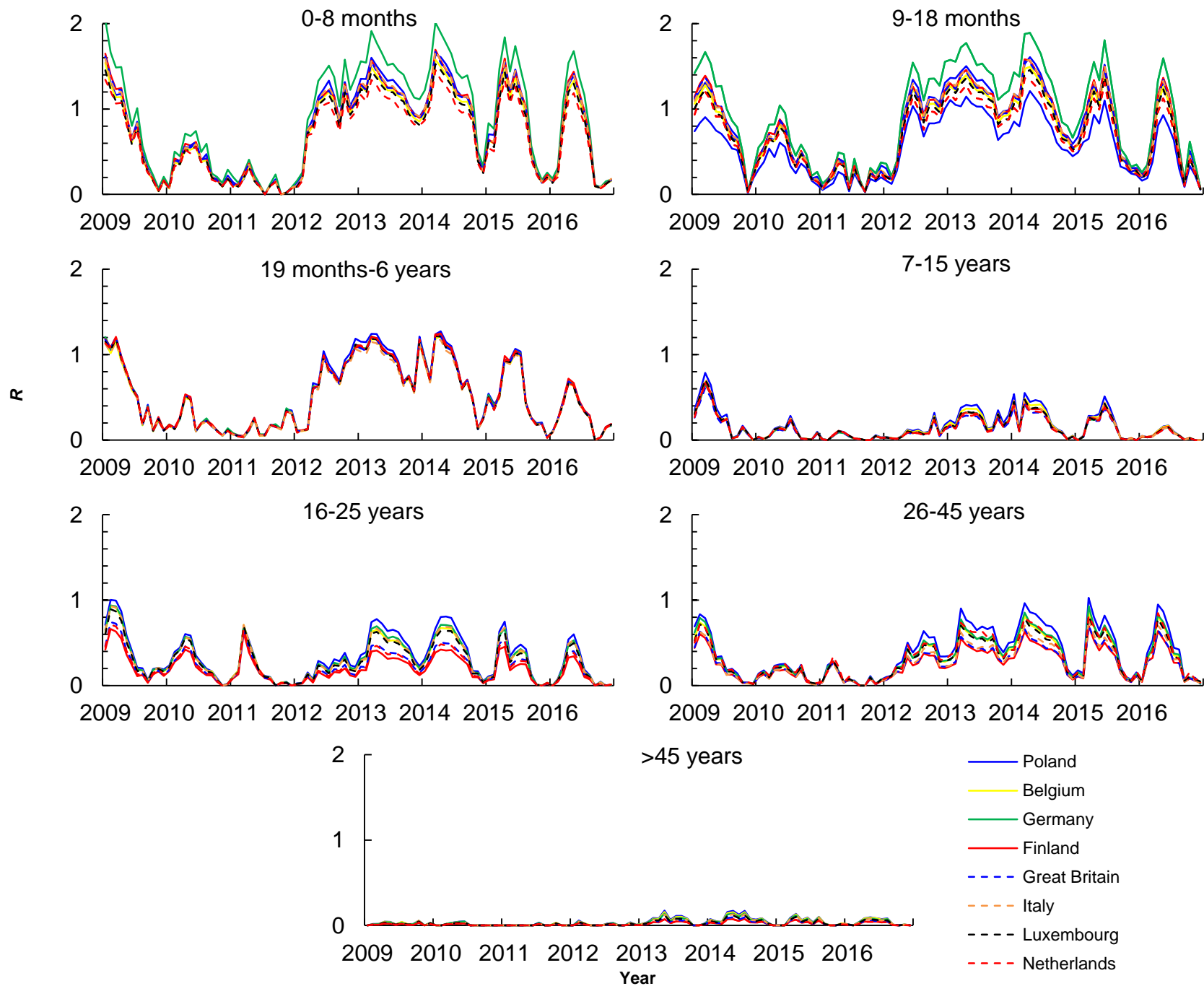

Supplement: S1 Fig — (PDF) [file pone.0205339.s001.pdf]
