# Supplementary material for: Monitoring the age-specificity of measles transmissions during 2009-2016 in Southern China
Source: PLoS One. 2018 Oct 8;13(10):e0205339. doi: 10.1371/journal.pone.0205339 (PMC6175510; doi:10.1371/journal.pone.0205339)
Supplement: S2 Fig — Monthly number of reported cases (left panel) and the estimated effective reproductive numbers (black line in right panel) with 1,000 realizations (grey lines in right panel) for the entire population and 7 age groups, using 12 days and 3 days as the mean and standard deviation of the gamma distribution of the serial interval. (PDF) [file pone.0205339.s002.pdf]

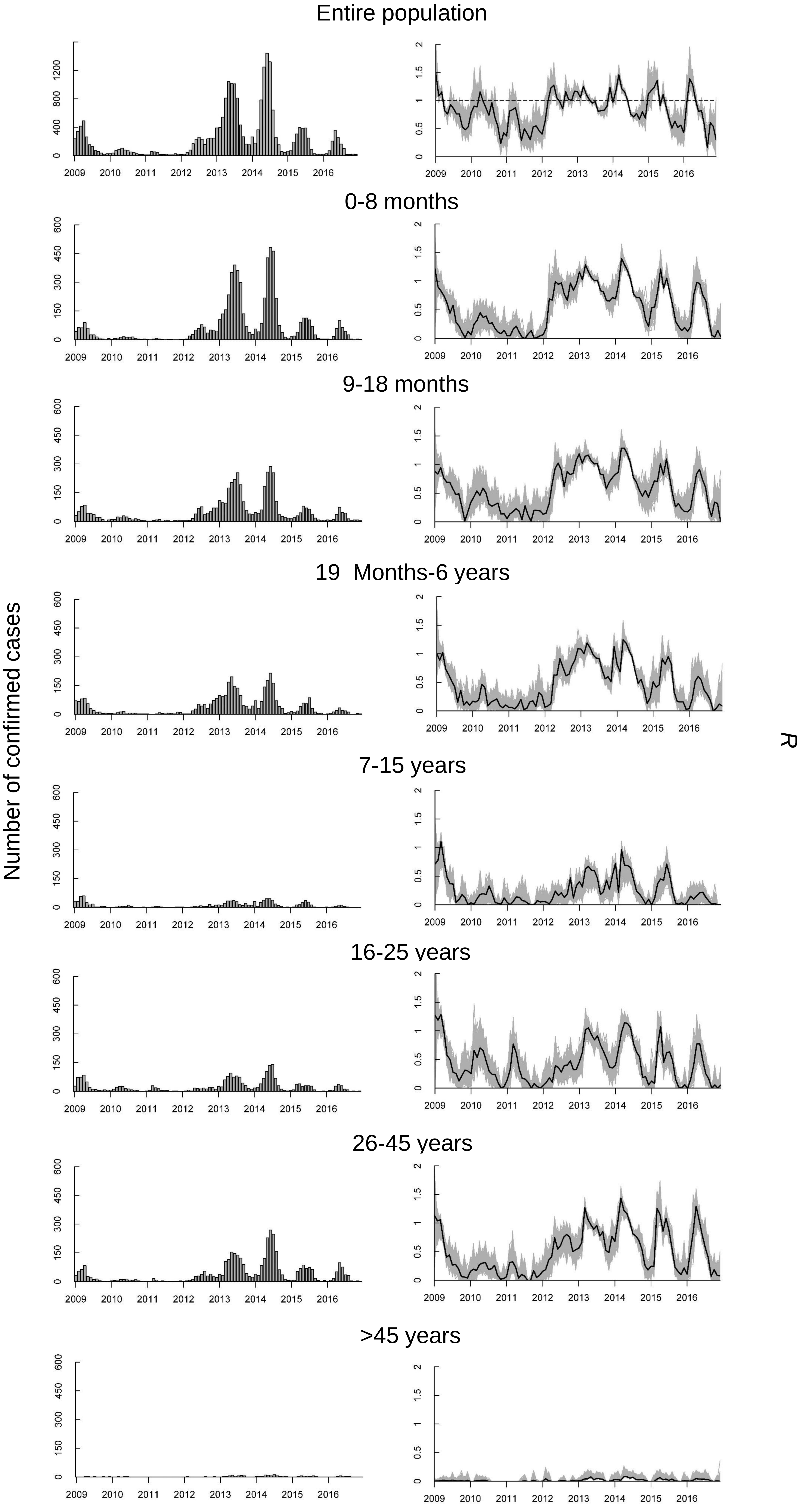
Entire population
0-8 months
9-18 months
19 Months-6 years
7-15 years
16-25 years
26-45 years
>45 years
Number of confirmed cases
R
2009
2010
2011
2012
2013
2014
2015
2016
